# Supplementary material for: Synthesis of a Rare Water-Soluble Silver(II)/Porphyrin and Its Multifunctional Therapeutic Effect on Methicillin-Resistant Staphylococcus aureus
Source: Molecules. 2022 Sep 15;27(18):6009. doi: 10.3390/molecules27186009 (PMC9501820; doi:10.3390/molecules27186009)
Supplement: Supplementary file 1 [file molecules-27-06009-s001.zip › molecules-1895782-supplementary.pdf]

molecules

MDPI

Supporting Information

Synthesis of a Rare Water-Soluble Silver(II)/Porphyrin and Its Multifunctional Therapeutic Effect on Methicillin-Resistant *Staphylococcus aureus*

Jiaqi He, Yu Yin, Yingjie Shao, Wenkai Zhang, Yanling Lin, Xiuping Qian and Qizhi Ren**

J. He, Y. Shao, Y. Lin and Ass. Prof. Q. Ren
School of Chemistry and Chemical Engineering, Shanghai Jiao Tong University, Shanghai 200240, P. R. China.
E-mail: qzren@sjtu.edu.cn

Y. Yin, W. Zhang and Ass. Prof. X. Qian
School of Pharmacy, Shanghai Jiao Tong University, Shanghai 200240, P. R. China.
E-mail: qianxp@sjtu.edu.cn

Contents

Figure S1. The Ag 3d XPS scan spectra of the centrifugal precipitate.

Figure S2. The ligand-field splitting diagram for the d^9 ion in a square-planar geometry.

Figure S3. The fluorescence lifetime spectrum of TMPPS in water (λ_{ex} = 414 nm).

Figure S4. The FT-IR full spectra of TMPPS and AgTMPPS.

Figure S5. Rate constant for ABDA absorbance change as a function of the laser irradiation time in the presence of AgTMPPS.

Figure S6. Time-dependent UV-vis spectra of ABDA, suggesting no $^1O_2$ generation by AgTMPPS without 460 nm light irradiation.

Figure S7. Rate constant for ABDA absorbance change at 378 nm as a function of the non-irradiated time in the presence of AgTMPPS.

Figure S8. The $^1O_2$ generation under 460 nm laser irradiation. (a) Time-dependent absorption of ABDA by methylene blue under laser irradiation. (b) Rate constant for ABDA absorbance change in the presence of methylene blue. (c) Time-dependent absorption of ABDA by AgTMPPS under laser irradiation. (d) Rate constant for ABDA absorbance change in the presence of AgTMPPS.

Figure S9. Time-dependent UV-vis spectra of AgTMPPS under laser irradiation (460 nm, 0.05 W/cm^2), suggesting the good photo-stability.

Figure S10. The redox process between 1,4-dihydronicotinamide adenine dinucleotide (NADH) and NAD^+.

Figure S11. Time-dependent UV-vis spectra change of the NADH+AgTMPPS group, suggesting no NADH depletion.

Figure S12. Time-dependent UV-vis spectra change of the NADH+Laser group, suggesting no NADH depletion.

Figure S13. The diagrammatic sketch of systematic antibacterial assays in vitro.

Figure S14. The schematic image illustrating the promising application of AgTMPPS, showing great bacterial disinfection potential in the biological field.

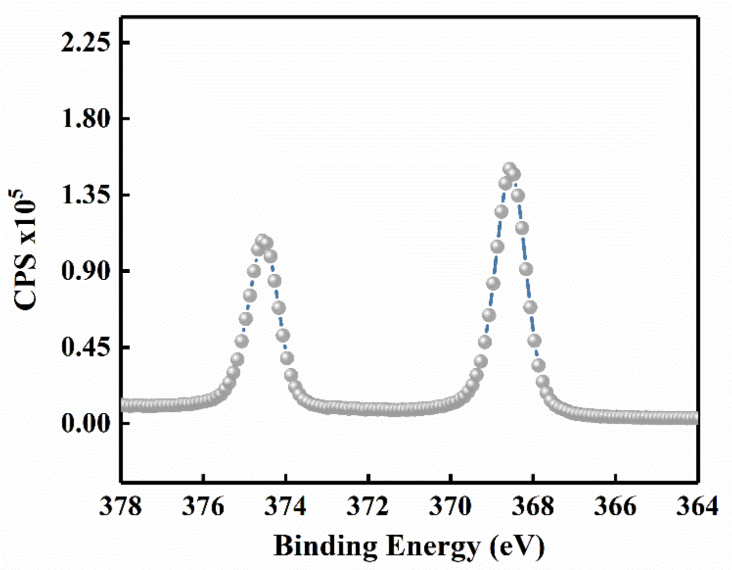

Figure S1. The Ag 3d XPS scan spectra of the centrifugal precipitate.

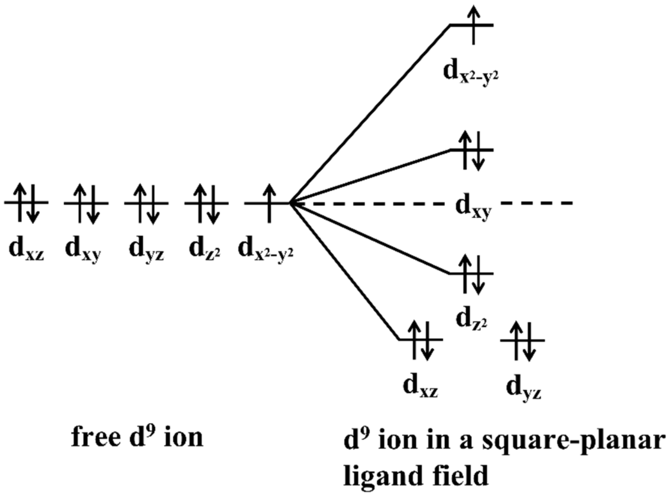

Figure S2. The ligand-field splitting diagram for the d^9 ion in a square-planar geometry.

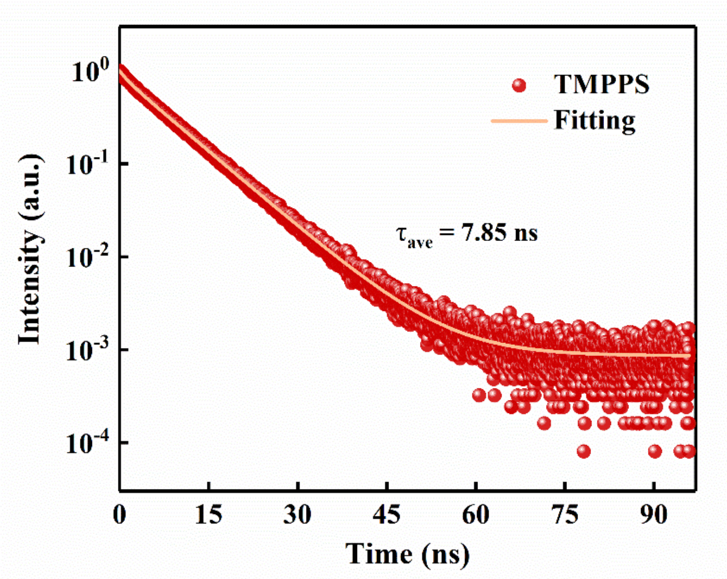

Figure S3. The fluorescence lifetime spectrum of TMPPS in water (λ_{ex} = 414 nm).

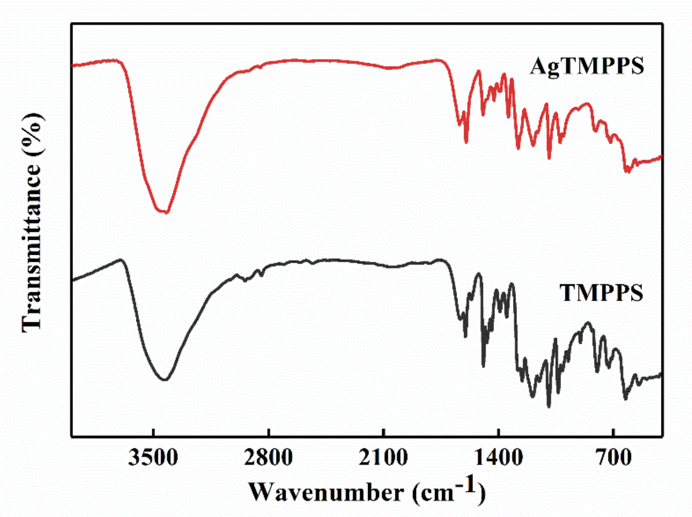

Figure S4. The FT-IR full spectra of TMPPS and AgTMPPS.

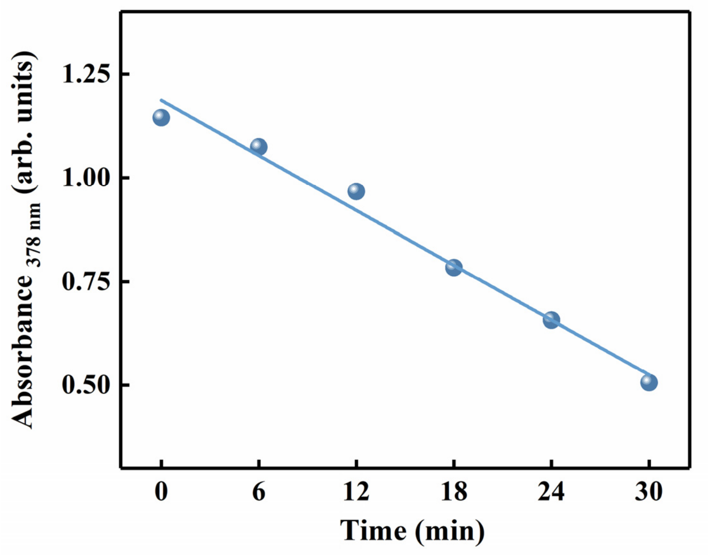

Figure S5. Rate constant for ABDA absorbance change as a function of the laser irradiation time in the presence of AgTMPPS.

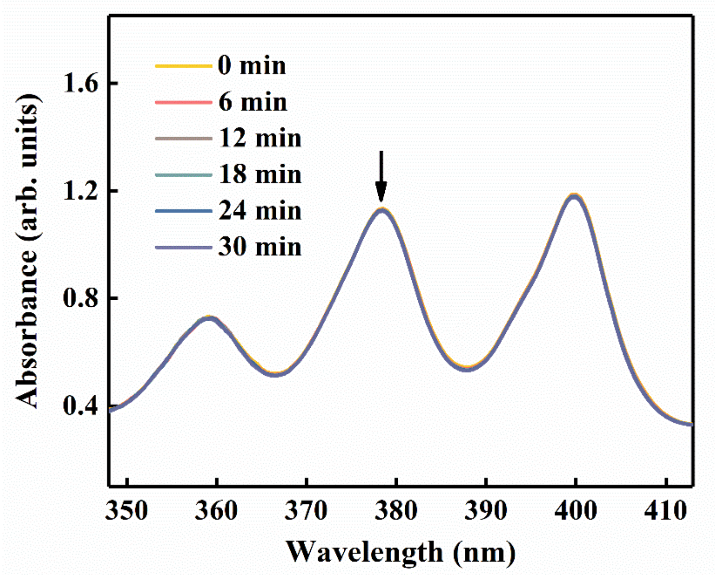

Figure S6. Time-dependent UV-vis spectra of ABDA, suggesting no $^1O_2$ generation by AgTMPPS without 460 nm light irradiation.

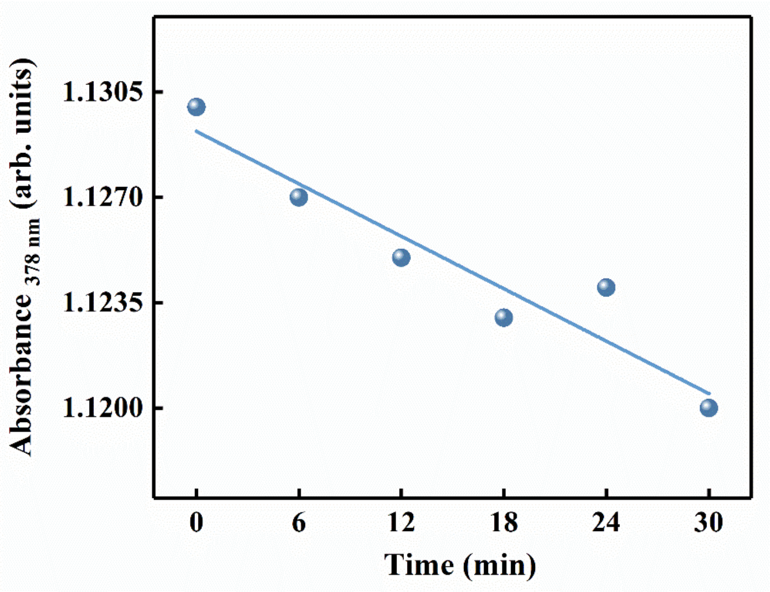

Figure S7. Rate constant for ABDA absorbance change at 378 nm as a function of the non-irradiated time in the presence of AgTMPPS.

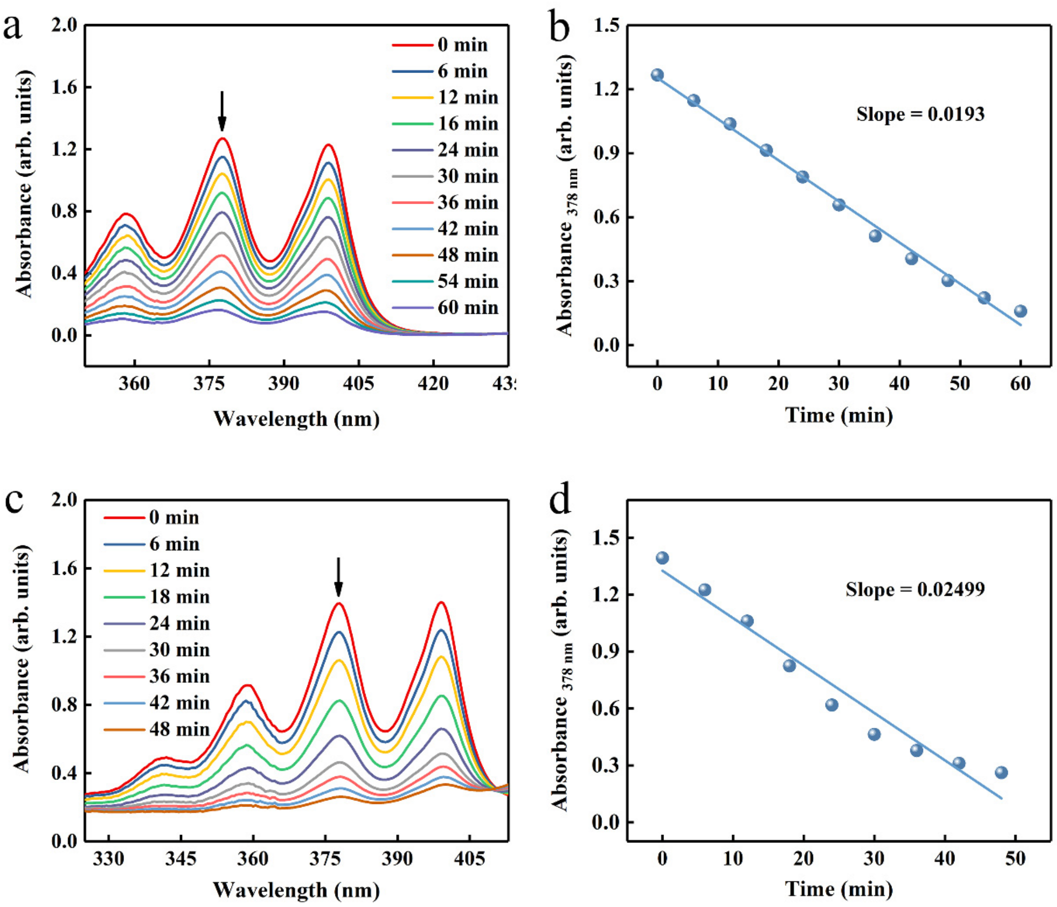

Figure S8. The $^1O_2$ generation under 460 nm laser irradiation. (a) Time-dependent absorption of ABDA by methylene blue under laser irradiation. (b) Rate constant for ABDA absorbance change

in the presence of methylene blue. (c) Time-dependent absorption of ABDA by AgTMPPS under laser irradiation. (d) Rate constant for ABDA absorbance change in the presence of AgTMPPS.

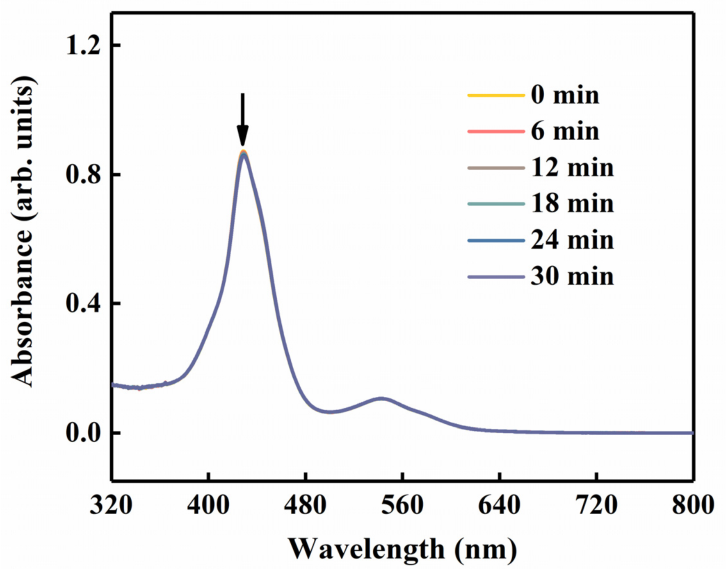

Figure S9. Time-dependent UV-vis spectra of AgTMPPS under laser irradiation (460 nm, 0.05 W/cm^2), suggesting the good photo-stability.

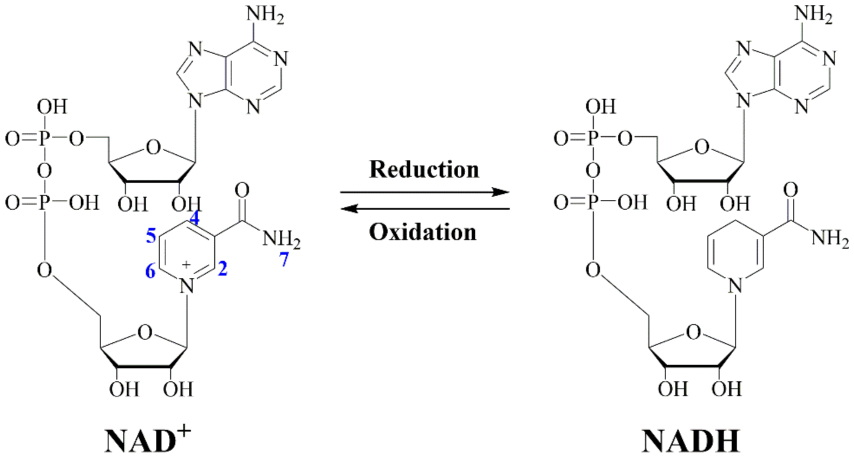

Figure S10. The redox process between 1,4-dihydronicotinamide adenine dinucleotide (NADH) and NAD$^+$.

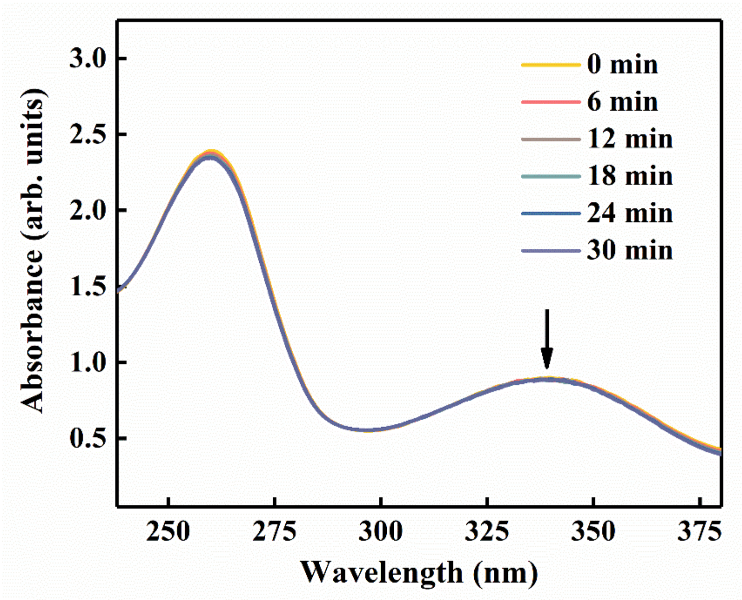

Figure S11. Time-dependent UV-vis spectra change of the NADH+AgTMPPS group, suggesting no NADH depletion.

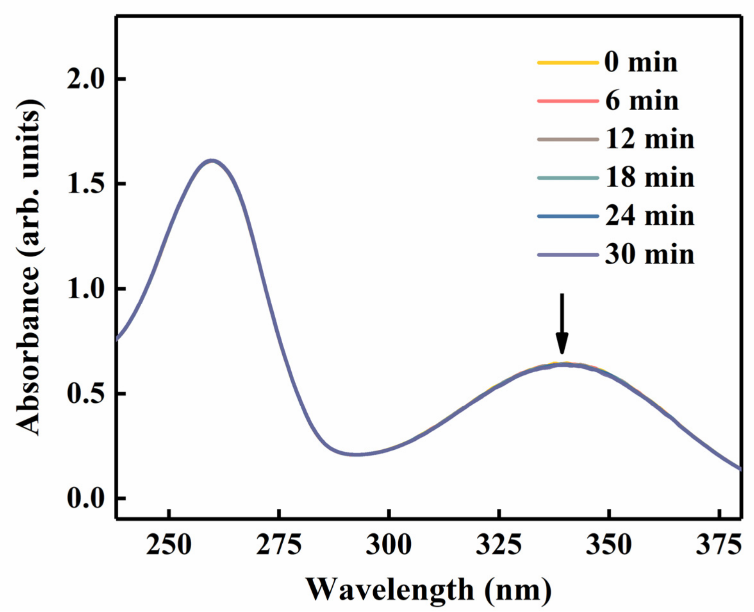

Figure S12. Time-dependent UV-vis spectra change of the NADH+Laser group, suggesting no NADH depletion.

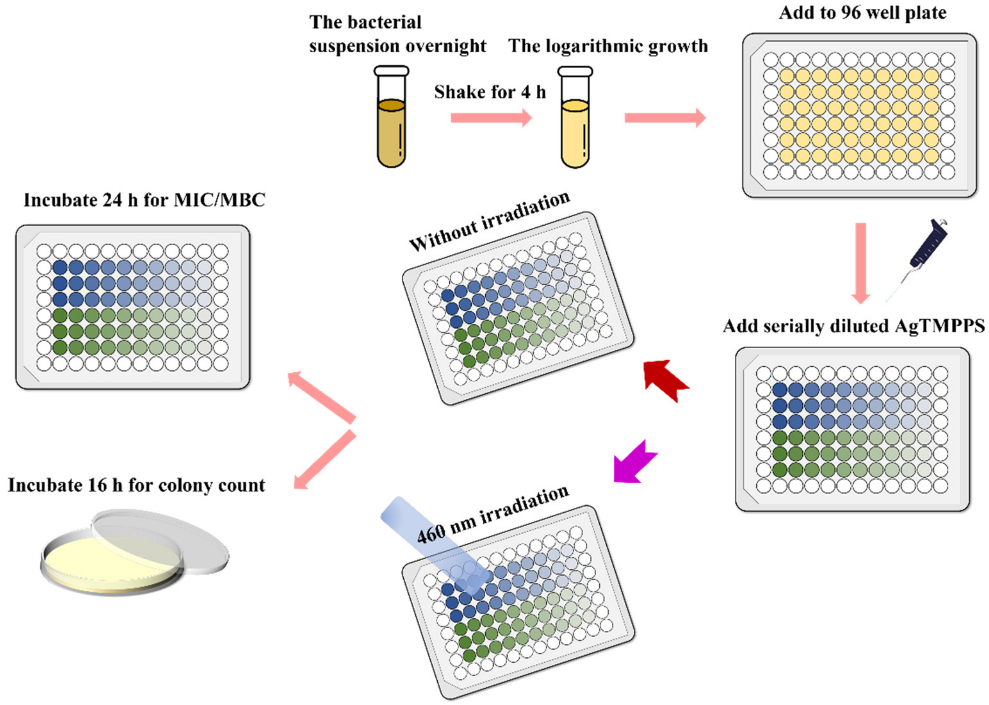

Figure S13. The diagrammatic sketch of systematic antibacterial assays in vitro.

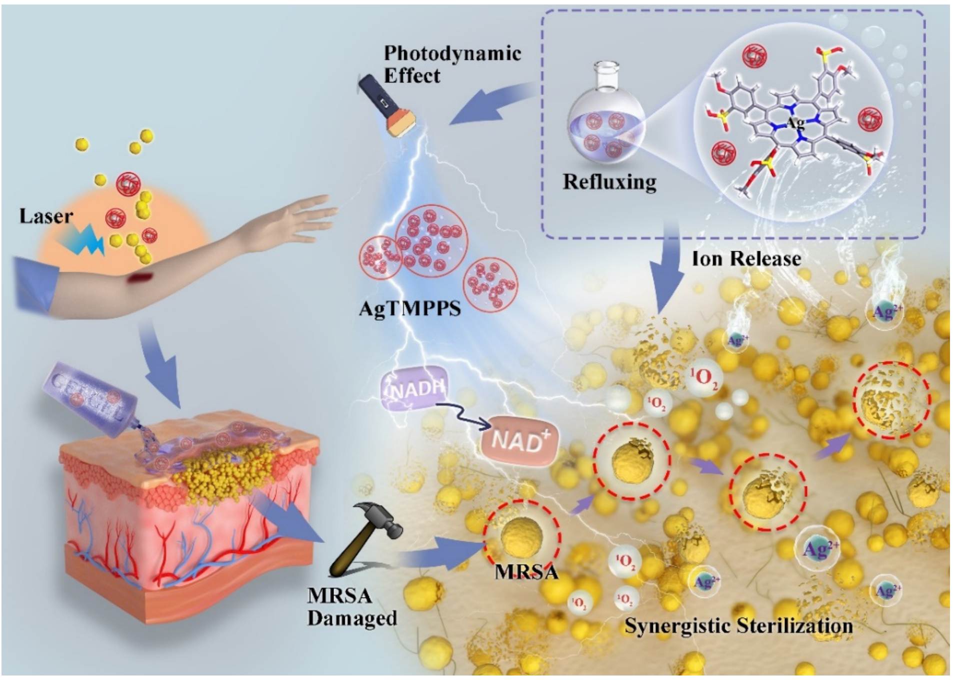

Figure S14. The schematic image illustrating the promising application of AgTMPPS, showing great bacterial disinfection potential in the biological field.
